# Benign mesenteric lipodystrophy presenting as low abdominal pain: a case report

**DOI:** 10.1186/1752-1947-4-119

**Published:** 2010-04-27

**Authors:** Jonathan Richard Rees, Phillip Burgess

**Affiliations:** 1 Department of General Surgery, Gloucestershire Royal Hospital, Great Western Road, Gloucester, GL1 3NN UK; 2 Department of General Surgery, Great Western Hospital, Marlborough Road, Swindon, SN3 6BB, UK

## Abstract

**Introduction:**

Benign mesenteric lipodystrophy is rare and often presents in a non-specific fashion. Imaging findings may mimic a range of malignant conditions, particularly malignant ovarian disease in women.

**Case presentation:**

We present the case of a 61-year-old Caucasian woman who was referred to the gynaecology service at our institution and was thought to have ovarian malignancy, and required a laparotomy. However, histopathological analysis unexpectedly revealed benign mesenteric lipodystrophy.

**Conclusion:**

Benign mesenteric lipodystrophy may mimic a range of conditions, particularly malignancy.

## Introduction

Benign mesenteric lipodystrophy is a rare condition with just over 200 cases being noted in the worldwide literature [1]. It was first described by Jura during the 1920s [2] and is characterized by non-specific inflammation involving the adipose tissue of the bowel mesentery [3]. It is commonly asymptomatic, or only noted during cross sectional imaging investigations that have been undertaken for other indications [4]. However, some patients present with symptoms that include abdominal tenderness or an abdominal mass while some may have abdominal pain, fever, a change in bowel habit and sometimes weight loss [5,6].

Its etiology is unclear. However, previous trauma, mesenteric ischemia or infection have been suggested as potential causes. Other documented possible associations with this disease include tuberculosis [1], pancreatitis, malignant tumour particularly lymphoma, vasculitis and granulomatous diseases [7]. The histopathology of this condition, which is also called sclerosing mesenteritis, has been described in three phases. Initially, fat necrosis is seen, leading to the nomenclature of mesenteric lipodystrophy. This is then followed by mesenteric panniculitis, which is associated with profound inflammation. Finally, fibrosis supervenes with mesenteric retraction and shortening [4], hence the term 'sclerosing mesenteritis'. In many cases, histopathology shows changes consistent with all three histopathological phases as these changes appear to occur at differing rates in different areas of the mesentery [5]. Patients with a treatable cause, such as tuberculosis, have been described to have almost complete resolution of the intra-abdominal change when re-imaged [1] particularly in the mesenteric lipodystrophy phase of the condition. We describe a case mimicking ovarian malignancy.

## Case presentation

**Figure 1:**
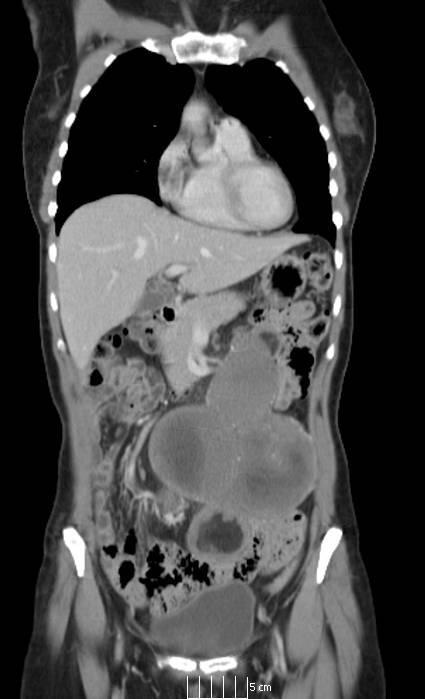
**Coronal computed tomography images showing multiple solid and cystic intra-abdominal lesions marked with an arrow with associated calcification in the bowel wall**.

**Figure 2:**
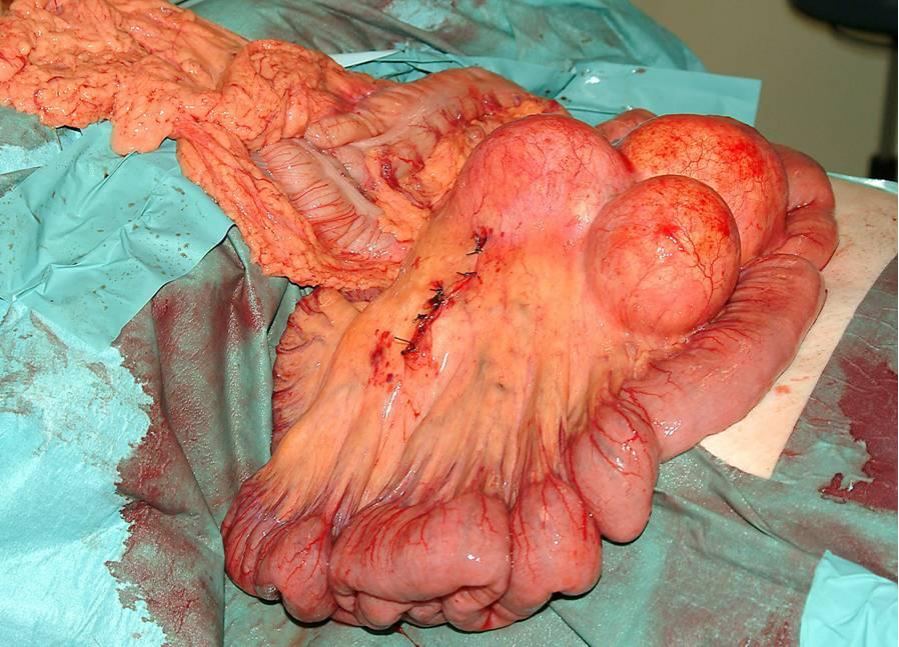
**Intra-operative photograph showing multiple small intestinal mesenteric masses**. Suture line represents site of operative sample of one of the multiple masses.

A 61-year-old British Caucasian woman was initially referred to the gynecology service in our institution with low abdominal and pelvic pain. An initial clinical examination was unremarkable. However, a pelvic ultrasound scan was undertaken and revealed cystic masses within the pelvis. A potential diagnosis of ovarian malignancy was considered and computed tomography (CT) was then performed, which also suggested an intra-abdominal mass of possible ovarian origin (Figure 1). In view of these findings, our patient underwent a laparotomy. At this time, the ovaries were noted to be normal. However, the mesentery of the small bowel was found to have multiple large mesenteric masses (Figure 2). These involved the majority of the small bowel mesentery and were irresectable. Biopsies were taken at this time and the mesenteric masses were shown to be benign with no evidence of lymphoma or epithelial malignancy, but were diagnostic for the mesenteric lipodystrophy stage if the illness. Our patient had an uncomplicated post-operative course and was discharged after five days.

A trial of tamoxifen 20 mg orally once daily was instituted in an attempt to reduce the size of the mesenteric masses. However, follow up CT of the abdomen did not shown any response after six months of therapy, so tamoxifen was discontinued. A follow-up small bowel study to exclude small bowel stricturing as a consequence of mesenteric fibrosis has not revealed any abnormality. Two years after the initial surgery, our patient remains well.

## Discussion

Benign mesenteric lipodystrophy is rare and has been described in most detail in a three-case series by Durst [6], Kipfer [7] and Emory [5] who together identify 165 patients. It is more common in men (2-3:1; M:F ratio), and affects a large age range from 20 to 80 years but is most common in individuals aged 50 to 60 years. It has a broad range of clinical presentations with at least half of those affected being asymptomatic. In these individuals, it is a usually found at the time of cross-section imaging, laparoscopy or laparotomy as in our case. The etiology of this condition is unclear. However, associations have been reported between mesenteric panniculitis and lymphoma [7] while an autoimmune etiology or as a response to ischaemia have also been postulated as possible causes [8,9].

A range of symptoms are described, including most commonly undiagnosed abdominal pain, and more commonly diagnosed signs of gastrointestinal obstruction. Fever, weight loss, abdominal mass or even a protein-losing enteropathy have been described. The frequency of differing symptoms is unclear because of the rarity of the condition but most reports suggest that the initial presentation is either with abdominal pain or as an asymptomatic finding at cross-sectional imaging. Biochemical tests are usually unhelpful, while hematological investigations may only show anaemia or a raised erythrocyte sedimentation rate (ESR) but are non-specific.

CT may help in making the diagnosis of mesenteric lipodystrophy. There are a number of features on CT that may suggest mesenteric lipodystrophy. These include increased attenuation in the small bowel described by Seo *et al*. [10] as the 'misty mesentery' or in more advanced cases there may be a solid soft tissue mass, which surrounds the mesenteric vessels with preservation of the surrounding fat around a "fat ring sign" on CT image [11,12].

The CT findings are unfortunately not specific and can mimic other lesions of the mesentery including lymphoma, lipoma, edema (of any etiology, for example heart failure, vasculitis, cirrhosis or hypoalbuminemia), tuberculosis, carcinomatosis or, very rarely, mesothelioma [10]. The extent of the change in the intra-abdominal fat may be wide-ranging and can include the mesocolon, mesoappendix, the peri-pancreatic region, the greater omentum and pelvic fat, which may explain why the differential diagnosis can be so extensive.

Diagnosis is usually during laparotomy, although it can also be done during biopsy at the time of laparoscopy or percutaneously [13,14]. Resectional surgery is of limited value in this setting [6]. Although smaller lesions may be resected for diagnostic purposes, the diffuse involvement of the mesentery would mean that excessively long segments of small bowel would have to be removed to clear the bulk of the mesenteric change resulting in significant morbidity.

Histologically, the condition shows a progressive series of changes. However, the different histological stages often co-exist within the same specimen. Initially, the mesentry is infiltrated with lipid-filled macrophages within the fat-filled septa of the mesenteric adipose tissue, which is known as mesenteric lipodystrophy. As the condition progresses and inflammation supervenes, lymphocytes infiltrate the mesentery and lipid cystic necrosis can be identified. This is a change known as mesenteric panniculitis. Later, necrosis with associated fibrosis dominates. This is associated with shortening of the mesentery, and is called the retractile mesenteritis stage. Typically, these changes may be identified by H&E staining. However, in cases where there is diagnostic difficulty particularly, when differentiating mesenteric lipodystrophy from gastrointestinal stromal tumours.(GISTs) and mesenteric fibromatosis, Montgomery *et al*. suggest that immunohistochemistry using a panel of antibodies (CD117, beta-catenin, CD34, smooth muscle actin, desmin, keratin, and S-100 protein) may help differentiate the histological types [15].

Treatment of this condition depends on the stage of the disease: early changes are nearly always managed conservatively as the illness resolves in many individuals with the lipodystrophy phase without intervention. In the later panniculitis or fibrotic phases of the illness, a range of treatments have been investigated. To suppress inflammation, steroids, cyclophosphamide [8], azathioprine and colchicine, treatment successes have also been reported after the use of tamoxifen and oral progesterones [16]. However, if fibrosis occurs that leads to symptomatic strictures of the gastrointestinal tract, then surgical resection of the affected segment is indicated [17,18].

## Conclusion

Mesenteric lipodystrophy is a rare condition that can mimic a number of intra-abdominal conditions including ovarian pathology. It can be difficult to diagnose and is often only fully apparent at the time of laparoscopy or laparotomy. Histologically, it forms part of a continuum of inflammation and fibrosis and may often in the early stages resolve spontaneously. Although often unnecessary in the early stages, treatment may require immunosuppression or even resectional surgery in the later stages if the disease progresses.

## Abbreviations

CT: computerised tomography; ESR: erythrocyte sedimentation rate; H&E: Haematoxylin and Eosin; GISTs: Gastrointestinal stromal tumours.

## Consent

Written informed consent was obtained from our patient for publication of this case report and accompanying images. A copy of the written consent is available for review by the journal's Editor-in-Chief.

## Competing interests

The authors declare that they have no competing interests.

## Authors' contributions

JR and PB were involved in the direct clinical care of our patient and therapeutic planning and both authors contributed equally to the manuscript. Both authors read and approved the final manuscript
